# Location-Enhanced Activity Recognition in Indoor Environments Using Off the Shelf Smart Watch Technology and BLE Beacons

**DOI:** 10.3390/s17061230

**Published:** 2017-05-27

**Authors:** Avgoustinos Filippoupolitis, William Oliff, Babak Takand, George Loukas

**Affiliations:** Computing and Information Systems Department, University of Greenwich, Old Royal Naval College, Park Row, London SE10 9LS, UK; william.oliff@gre.ac.uk (W.O.); b.takand@gre.ac.uk (B.T.); g.loukas@gre.ac.uk (G.L.)

**Keywords:** activity recognition, wearable devices, inertial sensors, Bluetooth beacons, machine learning

## Abstract

Activity recognition in indoor spaces benefits context awareness and improves the efficiency of applications related to personalised health monitoring, building energy management, security and safety. The majority of activity recognition frameworks, however, employ a network of specialised building sensors or a network of body-worn sensors. As this approach suffers with respect to practicality, we propose the use of commercial off-the-shelf devices. In this work, we design and evaluate an activity recognition system composed of a smart watch, which is enhanced with location information coming from Bluetooth Low Energy (BLE) beacons. We evaluate the performance of this approach for a variety of activities performed in an indoor laboratory environment, using four supervised machine learning algorithms. Our experimental results indicate that our location-enhanced activity recognition system is able to reach a classification accuracy ranging from 92% to 100%, while without location information classification accuracy it can drop to as low as 50% in some cases, depending on the window size chosen for data segmentation.

## 1. Introduction

Knowledge of context, with respect to the activity performed by a user, promotes the efficiency of human-centric technologies. Especially in an indoor setting, human activity recognition is beneficial for applications such as personalised health monitoring, building energy management, security and safety. Most activity recognition approaches use custom devices in order to gather data related to the activity performed. As we discuss in Section 2, these specialised devices are either worn on multiple body parts or are installed in various locations inside the building, forming a wireless sensor network. This can include a network of pressure, temperature, humidity and acoustic sensors installed in the area [1], proprietary sensors attached to objects within specific areas [2,3], optical monition capturing systems [4] and RFID tags [5].

These approaches, however, are obtrusive and suffer in terms of practicality, as multiple specialised devices have to be installed on various objects and in different locations inside the area. This also affects integration and user acceptance, as most of the times, these devices use communication protocols (e.g., ZigBee) that are not compatible with devices such as a mobile phone carried by a typical user. The goal of this research is to accurately recognise activities related to specific areas in an indoor space by only using commercial off-the-shelf devices and investigate the effect that information related to the user’s location has on the system’s performance.

To achieve this, we have designed and developed a system that is composed of a smart watch, BLE beacons, a mobile phone and a server. The system collects and processes data coming from these devices, without relying on specific or customised implementations, which results in enhanced flexibility. The popularity of wearable devices has significantly increased in recent years [6], while BLE beacons have become extremely popular, and there is a wide range of commercial offerings available from multiple manufacturers [7]. In previous work [8], we investigated the feasibility of activity recognition using commercial smart watches. Here, BLE beacons are used to enhance our activity recognition system in an unobtrusive way with information regarding the location of the occupants. In particular, in this work, we investigate our system’s performance when we fuse the inertial data coming from a commercial smart watch with data coming from BLE beacons. We have also evaluated different classification algorithms, feature types and segmentation window sizes. Our evaluation is based on real-world experiments, using our proposed system, that took place in an indoor laboratory environment.

In particular, the first contribution of this work is the development of an activity recognition system that incorporates commercial off-the-shelf BLE beacons in conjunction with wearable devices to enhance the system’s performance. As we discuss in Section 2, the majority of existing approaches either rely solely on wearable sensors or they use specialised infrastructure. The second contribution is the development of a data collection and labelling framework, which integrates the wearable devices and the BLE beacons and allows for the creation of labelled datasets to be used with activity recognition algorithms. Finally, the third contribution is the evaluation of our activity recognition system’s classification accuracy when using different classification algorithms and feature types and the comparison of its performance to that of systems that only rely on wearable devices.

We should note that the focus of this work is to evaluate the effect of location enhancement in recognising human activities. However, instead of only providing our experimental results for the location-enhanced system, we also present results for the case where only a wrist-worn device (i.e., a smart watch) is used by the participants. This provides the baseline that can help us compare the performance of the location-enhanced system to that of systems that only use wearable devices, as discussed in Section 2.

The remaining of this paper is structured as follows. In Section 2, we discuss related literature in the area of human activity recognition using wearable devices, both commercial and custom. We continue in Section 3 with a description of our system’s architecture, while Section 4 elaborates on the design of our activity recognition chain. The details of our experimental setup are presented in Section 5. In Section 6, we present our experimental results and discuss the performance of our system before we summarise our conclusions in Section 7.

## 2. Related Work

In the research field of Human Activity Recognition (HAR), there has been a growing trend of wrist-worn wearable devices (e.g., smart watches) with Inertial Measurement Units (IMUs) containing a host of different sensors. This was highlighted by a recent survey conducted in [9], which showed an even bigger trend in the use of IMUs with accelerometers, gyroscopes and magnetometers. The increased usage and interest of wrist-worn devices is not surprising, given the global acceptance of these devices in our daily lives. According to Statista [6], the number of worldwide sales for smart watches was five million in 2014, predicted to exceed 75 million in 2017, an increase of 1500% in three years.

There has also been a large amount of research conducted in the field of HAR that uses body-worn sensors, as highlighted by [10]. Body-worn approaches have been shown to have slightly better accuracy [11,12,13,14] than approaches that use only wrist-worn devices. However, body-worn approaches currently have the draw-back of being more obtrusive than their wrist-worn counterparts. Prolonged use of body-worn wearable devices may interfere with users’ daily lives, thus interfering with how the user performs activities. Moreover, body-worn wearables may become uncomfortable, limiting the devices to only being worn for short periods of time, rendering them less viable for monitoring daily activities. Wrist-worn approaches are less obtrusive and have less impact on the users’ daily lives, allowing the devices to be worn for longer periods of time. The literature presented is primarily focused on recent work that involves the use of wrist-worn wearables in the context of HAR.

We have provided some key details of related work in this area, as shown in Table 1. The details given in the table are:

- Indoor space: The type of indoor space the activity recognition experiments were carried out in, such as a home, a laboratory or another environment.
- IMU sensors: The different IMU sensors that were used by a classifier to infer a participant’s activity.
- Classification approach: What machine learning classification algorithms were used in the approach to estimate the activity performed by a participant.
- Commercial-Off-The Shelf (COTS): Whether the devices used in the experiments are widely accessible and available to an end user.

Furthermore, we classify captured activities into two general categories, low and high level activities. Low level activities are the ones that require whole body movement in order to be performed, such as walking, running and jumping. High level activities involve interaction with objects as described by [9] and daily activities being performed, as categorised by [10].

### 2.1. Activity Recognition Using COTS

A number of researchers are now incorporating COTS wrist-worn wearables into their approach for activity recognition rather than creating custom devices, as shown in Table 1. Additionally, COTS smart watches are usual paired with a smartphone, which also contains its own IMU. Therefore, this creates an optional data source that can be used if required, unlike their experimental wearable device counterparts.

The LG G Watch has been used in a couple of approaches [13,15], producing a reasonable level of accuracy. The authors in [15] use an LG G Watch coupled with a Samsung Galaxy S4 smartphone to identify eating activities for different foods. Their approach features a total of eighteen activities (six low and twelve high level) with seventeen participants. A range of classifiers (ANN with Multilayer Perceptron (MLP), NB and RF) are used, and the authors show that RF produces the best overall accuracy of 93.3% in personal classification. However, we should note that the authors do not provide evaluation for the other classifiers. Similarly, in [16], the authors propose a diet monitoring system that uses a smart watch device to detect fourteen eating and seven non-eating activities with a DT classifier. Using the accelerometer and gyroscope data from the smart watch, the authors show that the proposed system achieves an accuracy of 92% for detecting an eating episode. Furthermore, the authors in [13] use the accelerometer data from an LG G Watch for comparing activity estimation between sensor placement on the wrist and elbow (Myo armband) using KNN, DT, RF and bagging classifiers for eight high level gestures. The presented results show that the smart watch, with an accuracy varying between 86.5% and 96.2% depending on the classifier, does provide an 8% better overall accuracy over the armband, but not for every participant.

Another smart watch that has been shown to give good activity classification [17,18] is the Moto 360. The authors in [17] attempt to derive the activity of a shopper by capturing accelerometer and gyroscope data form a Moto 360 and a smartphone for high and low level activities respectively. Their approach achieves a precision accuracy of 92.26% for the high level activities when using an HMM and DT with Conditional Random Field (CRF) classifiers. The main aim in [18] is to increase the energy efficiency of a smart watch with the classification algorithm running locally. The authors not only use a Moto 360 (Motorola Mobility LLC., Libertyville, IL, USA), but also use a Samsung Galaxy Live (Samsung, Daegu, South Korea), Sony S3 (Sony Corporation, Tokyo, Japan) and LG G Watch R (LG Electronics, Seoul, Korea) for various evaluations throughout their work. The proposed system also provides a novel approach for semantic abstraction with NB, SVM, DT and LR classifiers, which delivers a good recall accuracy, averaging at 75% over the classifiers. However, it performs poorly in terms of precision accuracy, with the NB and DT classifiers producing 66% and 55%, respectively. Though, the authors do successfully demonstrate that semantic abstraction does improve overall accuracy.

Additionally, the researchers in [19] use a Microsoft Band smart watch for the purpose of identifying the activities of participants in a basketball game. A wide range of different classification algorithms (SVM, KNN, NB, DT and RF) is used. The authors use a personal classifier first used to distinguish the activity of the participant, and then, a collaborative classifier is used to identify the actual participant. With 10-fold cross-validation, the SVM classifier was shown to produce the best precision and recall accuracies of 91.34% and 94.31%, respectively.

The authors in [20] use the GENEActiv [21], which is a more specialised watch featuring a triaxial accelerometer and is geared towards research applications for free-living, sports research and clinical trials. The watch is available commercially and, therefore, still considered COTS technology. The approach in [20] is focused on classification of seven high level daily activities using HMM and CRF algorithms with leave-one-day-out cross-validation for the 21 days of data collected from two participants. It achieves accuracies ranging between 70% and 77% with the use of sub-classing and highlights that the GENEActiv is a feasible device for activity recognition.

A slightly more unorthodox approach is the one used by the authors in [22], who use two Samsung Galaxy S2 smartphones, with one at the pocket position and the other mounted at the wrist position. As a general rule, a smartphone weighs more than a smart watch. As a result, the device will potentially have a higher centre of gravity, which can affect how a participant performs activities. This aside, the authors in [22] do show that good classification accuracy of seven low level and six high level activities can be achieved using KNN, NB and DT classifiers. Additionally, they also show that using accelerometer and gyroscope data from the smartphone at the pocket position does improve the classification of static activities such as standing and sitting.

### 2.2. Activity Recognition with Custom Devices

There have been numerous works that use a custom made wrist-worn wearable device for the purpose of activity recognition. A custom wearable device removes any potential limitation that may be imposed by a COTS wearable device, such as adding additional sensors or collecting other information that a COTS wearable device API may not expose. Furthermore, HAR approaches that feature custom wearables devices commonly use additional on-body sensors or wearables to enhance activity classification performance.

One interesting use of a custom wrist-worn wearable device is the approach proposed in [23], where a wrist-worn device is coupled with wearable inertial rings to aid in increasing the accuracy of nine high level activities using DT and SVM classification algorithms. The approach is successful, as using only the wrist-worn device provides an accuracy of 68.85% for DT and 65.03% for SVM, while the whole system provides an accuracy of 89.06% for DT and 91.79% for SVM. Similarly, the authors in [24] use custom wearables at the elbow and wrist positions for the training phase for RF and CRF classifiers for smoking and eating sessions. For three high level smoking activities, the authors demonstrate that using the additional sensor at the elbow position results in an accuracy of 93% for RT and 95.74% for CRF. Furthermore, in [14], the authors evaluate and compare wrist- and body-worn sensors for DT, RT, NB, SVM and KNN classification algorithms with accelerometer data, in the context of fall detection. They show that RF achieves the best overall accuracy among the classifiers while the wrist worn device achieves 72% accuracy, which was marginally better than devices worn on other body locations such as the elbow and chest, which achieved 67% accuracy when classifying ten basic activities. However, sensors positioned at the ankle, knee and belt achieved an accuracy of 77%. This is also shown in the work conducted by [11], who compare hip and wrist sensor placements with an LR classifier using accelerometer data to classify seven basic activities. The authors demonstrate that the hip position provides better accuracy for four activities with an overall accuracy of 91%, while the wrist position provides better accuracy for the remaining three activities with an overall accuracy of 88.4%.

Other approaches using custom wearable devices at the wrist position exclusively include the work conducted by [25], who investigate how the combination of six classification algorithms (NB, SVM, DT, ANN with MLP, KNN and RF) can achieve better accuracy. The authors show that a combination of KNN and RF classifiers for four basic activities only using accelerometer data gives the best accuracy. Furthermore, the authors in [26] compare the ANN with MLP, NB and SVM classifiers using a custom wrist-worn wearable featuring a nine-axial IMU, showing the MLP-based ANN to be the best classifier for their approach. Similarly, the authors in [27] compare the performance of four classifiers (NB, ANN, DT and LR) for identifying eight basic sporting activities, when using a single wrist-worn custom wearable device fitted with a single accelerometer. They show that ANN is the best classifier, achieving an accuracy of 86.7%. The authors in [28] use Emerging Pattern (EP), which is a threshold classifier. EP has low computation requirements, allowing the authors to run the classification algorithm locally on the custom wearable device, which provided an overall accuracy of 86.2% when attempting to classify four basic activities. Lastly, in [29], the authors develop their own classification algorithm that is based on sign-of-slope and threshold evaluation to be used in conjunction with their custom wearable device featuring an accelerometer. They also compare their custom wearable against other COTS devices, specifically the iPhone 6 smartphone, Mi band and SKT smartbands and the Moto360 and Samsung Gear S smart watches. Though the authors’ approach is shown to provide better accuracy, it should be noted that data gathering for all of the COTS devices was performed simultaneously, with the participant holding the smartphone and wearing all four COTS devices. This could potentially prevent the participant from performing the activities under a real-life scenario due to the combined weight of the wearables.

### 2.3. Location-Enhanced HAR

The concept behind location-enhanced activity recognition is to use the location of a person as a feature of an activity. It is reasonable to assume that certain activities can only be performed in certain areas or locations. For example, in a home setting, food preparation would take place in the kitchen, while brushing your teeth would be performed in the bathroom. A recent survey conducted in [30] highlights how the location characteristic, as well as other characteristics (e.g., time, conditions, duration) of an activity can aid in the living of elderly people. Moreover, in [31], the authors show how having these additional characteristics can result in enriching activity modelling and recognition in providing assisted living in smart homes, resulting in activity classification estimates ranging from 88.26 to 100% for basic activities. Furthermore, gaining knowledge of a persons location can be used as an alternative method of improving activity classification as shown by [32], where the authors concluded that adding location awareness aides in activity recognition. Finally, the use of location-enhanced activity recognition grants the benefit of being a more unobtrusive approach as highlighted by [33] than other approaches that use more on-body sensors, as discussed in Section 2.2.

## 3. System Architecture

**Figure 1:**
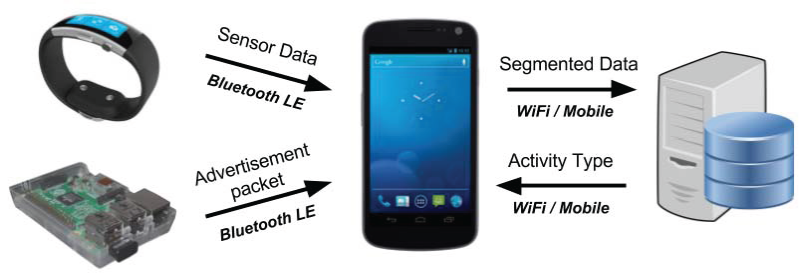
Overall system architecture.

Figure 1 illustrates the architecture of our system, detailing the inter-dependencies between its building blocks. Our system can accommodate any wearable device that provides an open API, while there is practically no limitation with respect to BLE beacons as we can adapt our approach to any commercial implementation.

To initiate the system operation, the user runs our mobile application on his/her smartphone, which begins gathering data from the smart watch and the BLE beacons. More specifically, the data are periodically read from the respective devices and transmitted back to the mobile phone using BLE. When the mobile phone has collected the necessary number of samples, which depends on the size of the segmentation window, it transmits them to the server, which uses a trained classifier to recognise the respective activity.

As processing takes place on the server, our system’s flexibility increases since we do not require mobile phones with high computational power or storage. The only requirement is to first conduct a data gathering phase, in order to build the dataset, which will be used for the supervised learning classification algorithms, as we further discuss in Section 4.

### 3.1. Smart Watch

There has been an increase in the popularity of wrist-worn sensors, such as smart watches and bands, in recent years. The majority of these devices use inertial sensors, such as accelerometers, in conjunction with health monitoring sensors, such as galvanic skin response and heart rate sensors. In this work, we have chosen to use the Microsoft Band 2. This is a “smart band” type of device and is equipped with a wide range of sensors, including three-axis accelerometer, gyrometer, optical heart-rate sensor, Galvanic Skin Response sensor (GSR), ambient light sensor, ultraviolet light exposure sensor and skin temperature sensor. The device offers a choice among three sampling periods for the accelerometer, namely 16, 32 and 128 ms. During our experimental procedure, we selected a sampling period equal to 32 ms, which results in a sampling frequency of 31 Hz approximately. This is appropriate for our application area, since the frequency required to assess daily physical activities is 20 Hz [34].

### 3.2. BLE Beacons

The requirement of being able to infer the location of people within a building has been a long-standing problem, mainly due to more traditional localisation services signals such as Global Navigation Satellite System (GNSS) being unable to reach the devices of users, especially in the context of large-scale buildings. BLE beacons have been successfully used in a wide range of location-aware applications, including remote healthcare monitoring [35,36], indoor navigation [37], tourism [38] and transportation [39]. Here, we decided to use a building occupancy detection approach [40,41,42].

This approach requires a reduced number of Access Points (AP) compared to other Indoor Positioning Systems (IPSs), resulting in a lower deployment cost and a more unobtrusive deployment. Furthermore, the battery life of these devices ranges from 6 to 24 months [7], which minimises maintenance requirements. Finally, the beacons use the BLE protocol, which is also used by most smart watch devices, and are able to communicate with the majority of mobile phones.

**Figure 2:**
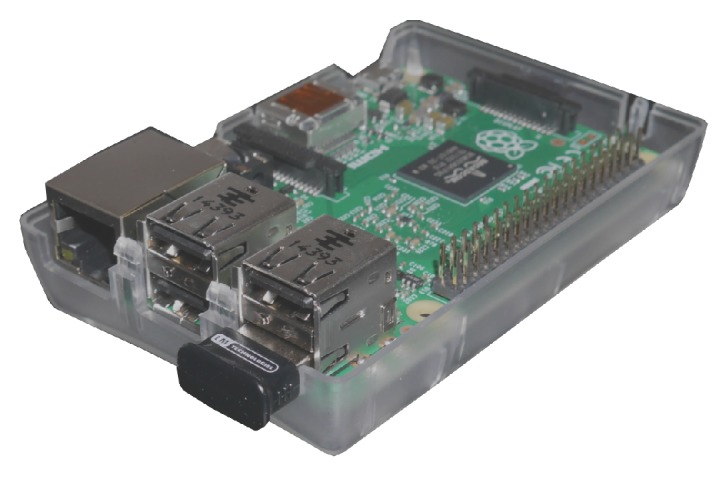
Raspberry Pi-based beacon.

To construct the BLE beacons for our system, we used off-the-shelf Bluetooth Low Energy (BLE) technology based on Apple’s iBeacon protocol. As shown in Figure 2, our beacons are based on a Raspberry Pi 2 Model B with an attached Bluetooth 4 LE module via a USB interface. The Raspberry Pis uses the BlueZ package to emulate a beacon and allow the customisation of the BLE advertising data being transmitted. Our beacons act as transmitters and broadcast a preset BLE advertising packet at set time intervals.

**Figure 3:**
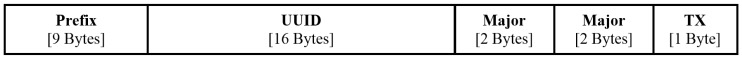
BLE beacon advertising packet structure.

To separate our beacons from other unassociated Bluetooth traffic and to be able to identify the beacons individually, a small hierarchy was introduced, which made use of the different identifiers available in the beacon packet structure, as illustrated in Figure 3. The Universally Unique Identifier (UUID) is used to define a universal group between all beacons; thus, giving the ability of being able to distinguish the BLE packets being used in our experiments from other Bluetooth traffic. The major number is used to define local groups of beacons who’s geographical locations are loosely connected. For example, beacons deployed on certain floors or buildings will have the same major number. Lastly, the minor number is used to identify each individual beacon within its local group.

### 3.3. Mobile Application

An Android mobile application was developed to gather the sensor data from the Microsoft Band and the BLE advertising data packets being broadcasted from the BLE beacons. During the system operation, the mobile device is paired with the Microsoft Band and receives the incoming sensor data stream. Furthermore, all BLE traffic the mobile device is in range of is being filtered, so only our beacon advertising data packets are being captured. Then, the respective beacon identifiers and the measured RSSI of each packet are stored. To filter out any unwanted data, the application firstly looks to see if the captured packet is structured in accordance to the iBeacon protocol by checking for the prefix (see Figure 3), and then, it will attempt to find the UUID being used by our beacons.

The application was designed with a modular approach, to allow it to be used with other wearable devices easily without the need to change the core program; thus granting the ability to integrate new wearable devices quickly and efficiently. During the data gathering phase of our experiments, a session is created for every participant. When performing a data capture, the activity about to be performed is selected from a drop down list. Then, during a data capture operated by the start/stop button on the application interface, sensor data from the smart watch and BLE advertising data from the beacons are collected simultaneously and stored locally before being sent to a server once the capture has finished.

### 3.4. Server

Mobile computing platforms have limited processing power and storage capacity compared to desktops and workstations. In particular, the processing power of wearable devices, such as smart watches and smart bands, is only adequate for their typical tasks, which include visual notifications, data collection and wireless communications. Smart phones offer improved processing power and memory capacity, but they still lag behind server-class computing solutions. To overcome these limitations, we have adopted a cloud-based solution for our system, which involves a server being responsible for the computationally-intensive tasks. More specifically, the role of the server is to process the data sent from mobile devices and then recognise the activity being performed. Initially, the classifiers that run on the server need to be trained using the data gathered during the data gathering phase. In normal operation mode, the server uses the trained classifiers to recognise the activities being performed by the users.

## 4. Activity Recognition Chain

**Figure 4:**
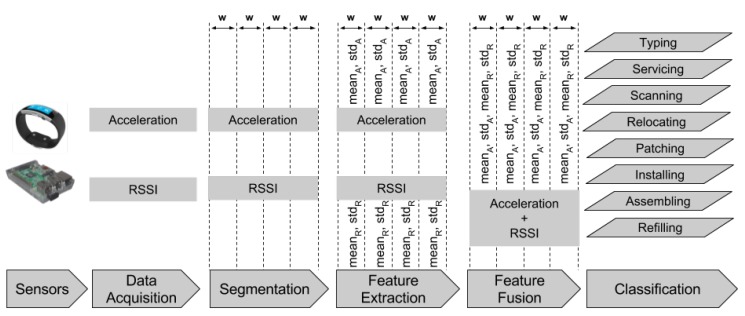
Overview of the activity recognition chain implemented in our system.

Figure 4 illustrates the procedure we followed to perform activity recognition. To simplify the illustration, we show the signal from one accelerometer axis for the smart watch and the RSSI from one beacon. During real-world operation, our system uses three accelerometer signals (one for each axis) and eight RSSI signals (one for each of the eight beacons deployed). For the smart watch, we have also experimented with both accelerometer and gyroscope signals, but this did not result in a noticeable improvement in performance.

The data acquisition phase is performed using our mobile application. When in training mode, where data need to be labelled for using them in the training of the classifiers, the participant has to select the activity he/she is performing from a list of available activities. This guarantees that incoming data will be labelled accordingly.

Our data segmentation approach involves the use of a non-overlapping sliding window. As we further discuss in Section 6.1, we have evaluated our system using window sizes of 1 to 5 s with a 1-s increment, as the window size has been shown to affect the performance of activity recognition [10,43,44]. We have used the same windowing mechanism for the BLE beacon data as it has been shown to benefit multipath mitigation [45].

With respect to feature extraction in the case of the accelerometer data from the smart watch, we have opted for two feature types:

- Type 1: mean and standard deviation.
- Type 2: mean, standard deviation, minimum, maximum and mean crossing rate.

These features are most appropriate for human activity recognition, as shown in the analyses in [43,46,47]. For the beacon data, we used one feature type, mean and standard deviation, based on our previous work on occupancy detection using BLE beacons [41,42]. As we are using a three-axis accelerometer, the total number of smart watch features for Type 1 is six, while for Type 2 it is 15. Similarly, since we have deployed eight beacons, the total number of beacon features is 16.

The next stage of our activity recognition chain is feature fusion [48,49]. In Section 6, we demonstrate that this significantly enhances the performance of our system. We must note, however, that our system can also operate using only the data coming from the smart watch accelerometers.

To better illustrate how feature fusion is implemented, let us define the RSSI signal value corresponding to beacon *i* at time *t* as: $r_{i}^{\left(t\right)}$, where i∈Z∩[1,K]. In our case, there are K=8 beacons.

Thus, at time *t*, the RSSI signal values corresponding to the eight beacons are: $r_{1}^{\left(t\right)}$, $r_{2}^{\left(t\right)}$, ... , $r_{8}^{\left(t\right)}$. Similarly, the accelerometer signal values for each axis at time *t* are: $a_{x}^{\left(t\right)}$, $a_{y}^{\left(t\right)}$, $a_{z}^{\left(t\right)}$.

In the data segmentation stage, the signals from each sensor are partitioned into non-overlapping data windows $w_{s}$, where *s* denotes the type of sensor. Consequently, we have:

$$\begin{array}{cc} w_{r_{1}} & =(r_{1}^{\left(t_{1}\right)},...,r_{1}^{\left(t_{n}\right)})\\ ...\\ w_{r_{8}} & =(r_{8}^{\left(t_{1}\right)},...,r_{8}^{\left(t_{n}\right)})\\ w_{a_{x}} & =(a_{x}^{\left(t_{1}\right)},...,a_{x}^{\left(t_{m}\right)})\\ w_{a_{y}} & =(a_{y}^{\left(t_{1}\right)},...,a_{y}^{\left(t_{m}\right)})\\ w_{a_{z}} & =(a_{z}^{\left(t_{1}\right)},...,a_{z}^{\left(t_{m}\right)}) \end{array}$$

We must note that, since the transmission frequency of the BLE beacons and the sampling rate of the smart watch are different, the number of samples in the respective windows also differ, as denoted by $t_{n}$ and $t_{m}$. For each window, we extract a set of features, which are then fused into a single feature vector x. For example, if we use the first feature type (mean and standard deviation) for the smart watch data, the fused feature vector for K=8 beacons will be:

$$\begin{array}{c} x=(mean\left(w_{r_{1}}\right),std\left(w_{r_{1}}\right),...,mean\left(w_{r_{8}}\right),std\left(w_{r_{8}}\right),\\ mean\left(w_{a_{x}}\right),std\left(w_{a_{x}}\right),mean\left(w_{a_{y}}\right),std\left(w_{a_{y}}\right),mean\left(w_{a_{z}}\right),std\left(w_{a_{z}}\right)) \end{array}$$

The feature vector x is then used as the input to the classifier. For the classification of activities, we have chosen four classifiers that have been successfully used in human activity recognition research, as discussed in Section 2. More specifically, we have chosen k-Nearest Neighbours (KNN), Logistic Regression (LR), Random Forest (RF) and Support Vector Machines (SVM). We partitioned our dataset into 80% training set and 20% test set and used 10-fold cross-validation for hyper-parameter tuning. For SVM, we have chosen the radial basis function kernel, as the number of features is small compared to the number of instances, and mapping our data to a higher dimensional space improves the classification performance [50].

We should note that when the system is used in normal operation mode with a trained classifier residing in the server, as depicted in Figure 1, the mobile phone is responsible for the stages up to and including segmentation. The data are then transmitted to the server where feature extraction, fusion and classification take place.

## 5. Experimental Setup

In this section, we present the approach we adopted for deploying our BLE beacons and conducting our activities. We first give the details of the indoor space where our experiment took place. We then elaborate on the types and durations of activities performed.

### 5.1. Beacon Deployment

**Figure 5:**
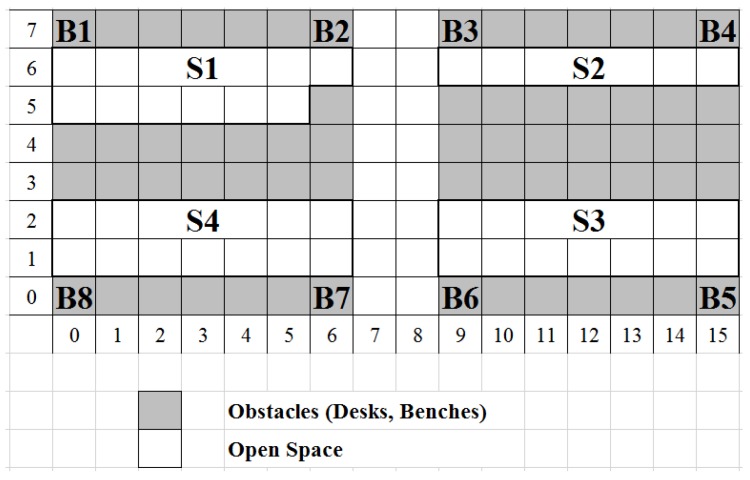
Beacon deployment.

Eight beacons were deployed inside the University of Greenwich computer laboratory. We have used a virtual grid to map the experimental area and illustrate the geographical positions of the beacons, as depicted in Figure 5.

Each grid block represents a 1 m × 1 m area. Grey blocks represent an area that a participant cannot reach (in terms of location) due to an obstacle, whereas the white blocks represent accessible area to the participants. Additionally, the floor plan shows the four sectors within the laboratory. Sectors 2 to 4 are computer bays, and Sector 1 is the technical support staff laboratory area. Throughout the experiments, all of the beacons were configured with an advertising data packet frequency of 7 Hz. This deployment density, as our previous work [41] has shown, provides sufficient performance with respect to location accuracy.

### 5.2. Laboratory Activities

Using our framework described in Section 3, we collected data for eight different activities that would be typically performed by a technical support staff member. Table 2 illustrates the relationship between area sectors (shown in Figure 5) and activities that can take place inside each sector.

**Figure 6:**
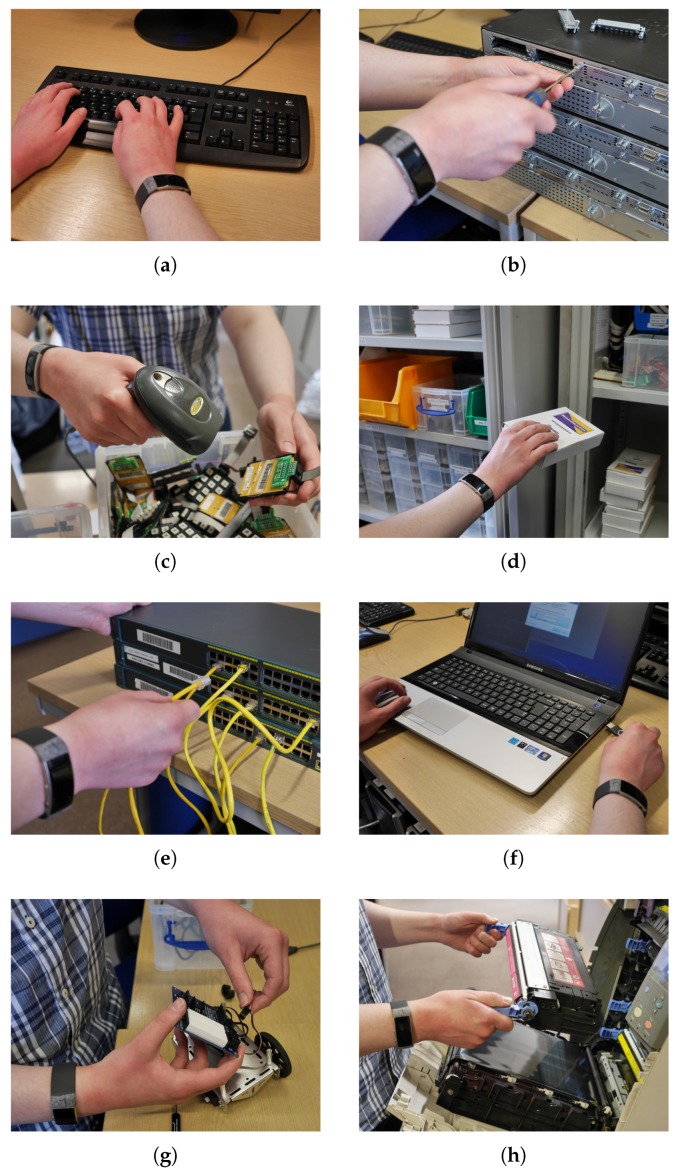
The activities performed by the participants in our laboratory. (**a**) Typing; (**b**) servicing; (**c**) scanning; (**d**) relocating; (**e**) patching; (**f**) installing; (**g**) assembling; (**h**) refilling.

This mapping is based on the layout of the experimental area and provides an increased level of realism to our experimental process. For example, refilling printer cartridges takes place in Sectors 1 and 2, since this is the location of the two printers, while the scanning activity only takes place in Sector 1, as this is the location of the barcode scanner. We should also note that each activity was performed in different locations within the same sector, among participants and repetitions. For example, the network switch during the patching activity was positioned in various locations along the benches inside Sectors 2, 3 and 4. Figure 6 illustrates the activities being performed by a participant, while a detailed description of each of the activities is given below:

- Typing: When conducting this activity, the participants used a standard desktop-style computer located in the laboratory, as depicted in Figure 6a. The computer was prepared with randomly-chosen excerpts at the top of the screen with a word processing application at the bottom of the screen. Then, the participants simply needed to type the text into the word processor.
- Servicing: In this activity, the participants were performing servicing tasks on computer equipment by removing and replacing service panels and changing over individual components. This is illustrated in Figure 6b. More specifically, the participants were exchanging components in the network router units by unscrewing the service panels.
- Scanning: For this activity, depicted in Figure 6c, the participants were asked to scan large amounts of small embedded components (LCD screens, keypads, sensors units, etc.) with applied bar codes using a hand-held scanner. This activity would be typically performed when loaning equipment to students or staff or when taking a stock check. Additionally, the participants were only asked to use their dominant hand to hold the scanner when performing this activity.
- Relocating: This activity consisted of moving large volumes of equipment from one storage location to another, as shown in Figure 6d. When performing this activity, the participants were only told to move one piece of equipment at a time. All equipment relocated by the participants could be grasped using only one hand.
- Patching: Within this activity, the participants were presented with multiple network switches accompanied by enough Ethernet cables to be inserted into every port of the switches. Figure 6e illustrates this setup. Each participant was instructed to patch in the Ethernet cables across the multiple switches in any way he/she wished. Additionally, the supplied Ethernet cables were not of equal length.
- Installing: This activity involved the installation of various software packages on a laptop, as shown in Figure 6f. Moreover, the laptop was turned on and was prepared with none of the software packages installed. Then, each participant was supplied with a USB flash drive containing the installers for the software packages and was only instructed on the order in which the packages should be installed.
- Assembling: When conducting this activity, depicted in Figure 6g, the participants were presented with a small dismantled vehicular robot with brief assembling instructions and a basic toolkit. Only required parts and tools were supplied; no additional equipment was given. The only instruction given to each participant was to assemble the robot using the tools and instructions provided.
- Refilling: In this activity, the participants were performing maintenance on two printers located in the laboratory. More specifically, as Figure 6h illustrates, the participants were asked to replace the various printer cartridges. To perform this activity, the participants were required to open the service panel of the printer and then replace the old cartridge with a new cartridge. Finally, the participant would close the service panel of the printer. No tools were required to open and close the service panel of the printer.

Our analysis focuses on recognising activities that a technical support staff member would perform and how this process can be enhanced by location information. There are, however, other activities that the participants can perform before or after they engage in one of the activities we described above. As the set of these activities depends on the context and the environment in which the system operates, we would expect that inside a computer laboratory, a participant could also be walking, standing still, sitting on a chair, etc. Our system can be adapted in order to address this. One approach we can adopt is to expand our training dataset to include a wider range of activities. This would result in a higher number of classes in our multiclass classification problem. Another approach is the inclusion of the null class, which is formed by activities that have similar patterns, but are irrelevant with the application in question. However, since in theory there is an infinite number of arbitrary activities that can belong to the null class, modelling it is particularly difficult [43].

The data collection was carried out by using our mobile application in training mode. Data coming from the smart watch and the BLE beacons were logged by the mobile application. Furthermore, when participants were performing activities, they were only given the required basic information to minimise the amount of external influence on the participant. This allowed us to perform the activities under a more naturalistic setting, closer to real-life conditions.

Each of the aforementioned activities was performed for a time between 170 s and 180 s by three different participants, while two out of three participants repeated the activities one more time. This resulted in a total dataset duration of about 290 min.

## 6. Results

In this section, we present the results of our activity recognition system. We have evaluated the system in a wide range of window sizes, classification algorithms and feature types, as discussed in Section 4. We first provide an overview of our system’s performance for different windows sizes and feature types and continue with an evaluation of individual activities. In order to provide a comparison with the methods that only use COTS or custom wearable devices, as discussed in Section 2, we have also evaluated the performance of our system when only using data generated by the smart watch. As the main focus of this work is to demonstrate that location enhancement significantly benefits activity recognition, these results form the baseline against which we measure the enhancement in system performance when incorporating BLE beacon data.

### 6.1. Overview of Results

We begin with providing a high level view of our system’s performance over the range of different parameter values. A first metric we used for our evaluation is the $F_{1}$ score [51], which takes into account both precision and recall and is robust to class imbalance. It is defined as:

(1) $$F_{1}=2\times \frac{precision\times recall}{precision+recall}$$

where $F_{1}\in \left[0,1\right]$, $precision=\frac{tp}{tp+fp}$, $recall=\frac{tp}{tp+fn}$, tp=truepositives, fp=falsepositives and fn=falsenegatives. A value of $F_{1}$ close to one indicates the best classification performance.

**Figure 7:**
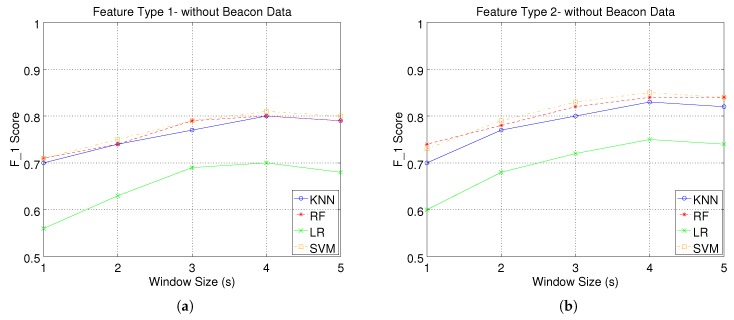
Activity recognition system performance without beacon data: weighted average of $F_{1}score$ over all activities, for different window sizes, feature types and classification models. (**a**) Wearable Feature Type 1 (mean, standard deviation); (**b**) Wearable Feature Type 2 (mean, standard deviation, mean crossing rate, maximum and minimum).

**Figure 8:**
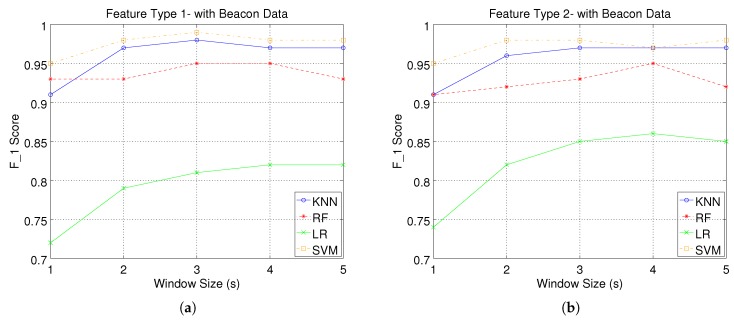
Activity recognition system performance with beacon data: weighted average of $F_{1}score$ over all activities, for different window sizes, feature types and classification models. (**a**) Wearable Feature Type 1 (mean, standard deviation); (**b**) Wearable Feature Type 2 (mean, standard deviation, mean crossing rate, maximum and minimum).

Figure 7 and Figure 8 present the $F_{1}$ score performance of our system. We must note that we have calculated the weighted average of $F_{1}$ score over all activities, weighted by the number of true instances for each class, for different window sizes, feature types and classification models.

More specifically, in Figure 7a, we illustrate our system’s performance when using the first feature type without beacon feature fusion. We can observe that LR performs considerably worse compared to the other three classifiers. More specifically, KNN, RF and SVM are all able to achieve a maximum $F_{1}$ score of 0.8 for a window size of 4 s, while LR achieves a $F_{1}$ score of 0.7 for the same window size. Increasing the window size improves the classification performance; however, exceeding a size of 4 s does not yield further improvement. The same performance pattern can be seen in Figure 7b, where there is a significant performance gap between LR and the rest of the classifiers. Both figures indicate that using a higher dimensional feature space for the smart watch data (Feature Type 2), improves the performance of all classifiers.

Figure 8a,b presents our system’s performance when using BLE beacon data in conjunction with smart watch data. It is evident that there is a significant enhancement in the system’s performance as illustrated by the improved $F_{1}$ scores for all classifiers. We should note that when using location-enhancement, all classifiers, except LR, are able to achieve $F_{1}$ scores above 0.9 even for the smallest window size of 1 s. As a small window size improves our system’s response time (less time required to recognise the performed activity), this result highlights the benefit of using beacon feature fusion in our activity recognition system. We can also observe a similar performance pattern for the case where no beacon data are used, both with respect to window size and to the gap between LR and the rest of the classifiers. However, we should note that now there is a more clear distinction among the classifiers in terms of performance. SVM achieves the best $F_{1}$ score for all experimental configurations, followed by KNN and RF, respectively.

**Figure 9:**
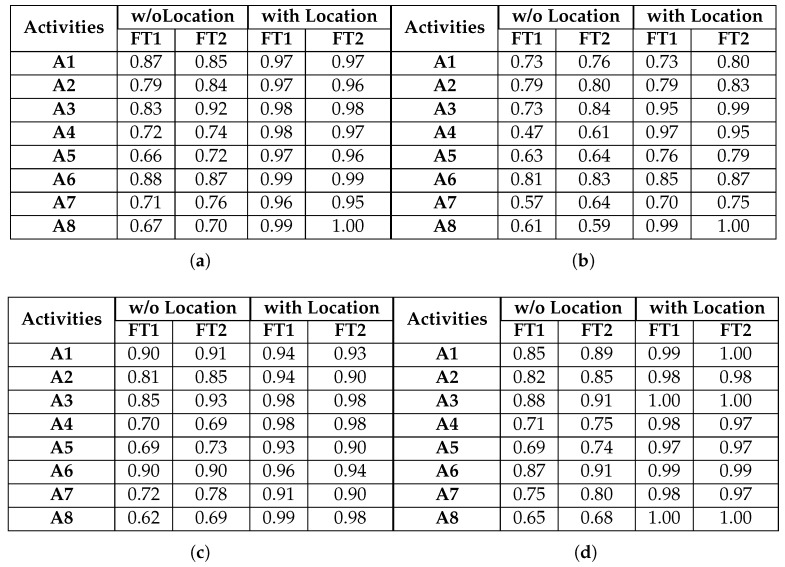
$F_{1}$ Scores for all classifiers and activities, for a window size of 3 s. (**a**) KNN; (**b**) LR; (**c**) RF; (**d**) SVM.

An overview of activity-specific classification performance across all experimental configurations is illustrated in Figure 9. We can again confirm that the performance pattern observed in Figure 7 and Figure 8 is present: LR has a consistently worse performance compared to the other classifiers; increasing the smart watch feature dimensionality improves classification performance; and beacon feature fusion significantly enhances classification performance for all activities and classifiers.

### 6.2. Evaluation of Individual Activities

The observations of Section 6.1 have informed the choice of system parameters that are investigated here, where we elaborate on our system’s performance for individual activities. Based on these observations, window sizes higher than 4 s do not significantly benefit the system’s performance. Furthermore, there is a clear performance gain when using the second feature type for the smart watch data. Thus, we analyse individual activity classification for window sizes up to 4 s when using the second smart watch feature type, with and without beacon feature fusion. We will refer to the activities with the codes assigned in Table 2.

To better illustrate our system’s performance, we present our results for each classifier using a normalised confusion matrix. A row of the matrix represents the instances in an actual class, while a column represents the instances in a predicted class. The diagonal elements represent the number of instances where the predicted label is equal to the true label. Off-diagonal elements represent instances that are misclassified. Furthermore, we have normalised the confusion matrices by the number of elements in each actual class. In the case of class imbalance, this approach better illustrates which classes are being misclassified. Furthermore, we have colour-coded the matrices by assigning black to 1.0 (100%) and white to 0.0 (0%). Finally, we should emphasise that the evaluation results have been calculated using only the test set data (20% of the original dataset), which the classifiers have never seen before, in order to provide a more reliable estimation of their out of sample error.

**Figure 10:**
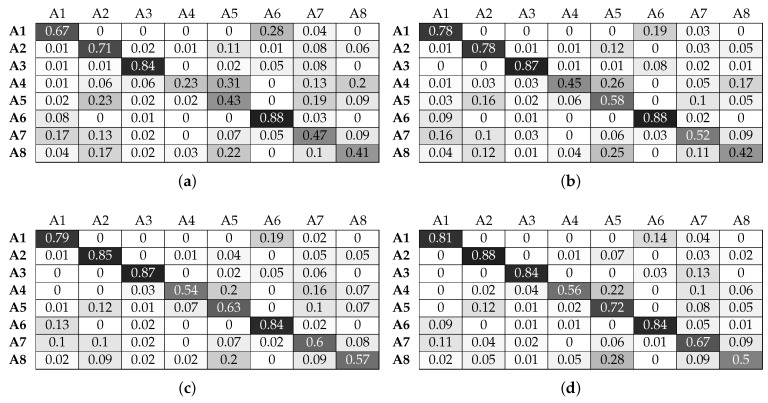
Normalised confusion matrices for logistic regression, with Wearable Feature Type 2, without beacon data. (**a**) C = 10, w = 1 s; (**b**) C = 100, w = 2 s ; (**c**) C = 10, w = 3 s; (**d**) C = 10, w = 4 s.

**Figure 11:**
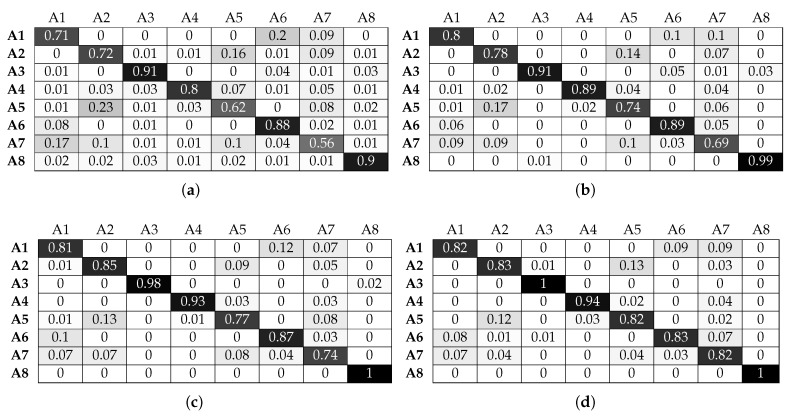
Normalised confusion matrices for logistic regression, with Wearable Feature Type 2, with beacon data. (**a**) C = 10, w = 1 s; (**b**) C = 10, w = 2 s ; (**c**) C = 10, w = 3 s; (**d**) C = 100, w = 4 s.

As shown in Section 6.1, the LR classifier results in the lowest classification performance in all experimental configurations. Figure 10 and Figure 11 confirm this observation for individual activities. More specifically, for activities A1, A2, A3 and A6, the LR classifier without beacon information is able to achieve a classification accuracy that increases with window size and manages to reach 80%. Adding beacon information does not significantly change the performance of the classifier for activities A1, A2 and A6. However, A3 benefits significantly and reaches 100% accuracy for a window value of 4 s. Looking at Table 2, we can see that A3 takes place in Sector 1, while A1, A2 and A6 do not. This is beneficial for the classification and allows the LR classifier to better distinguish between the activities. We must note that, although A6 can also take place in Sector 1, the actual micro-location within this sector is different (scanning and installing take place in subtly different locations along the bench). This is adequately different for the classifier to improve its performance. Looking at activities A4, A5, A7 and A8, we observe that LR gives poor classification performance without beacon data. For example, A4 is misclassified as A5, with more than 50% of examples classified incorrectly. More specifically, we can note that the activities of patching and relocating both involve translational hand movement while grasping an object (a cable or a piece of equipment). This can be confirmed by Figure 6d,e.

To further explain this, we must note that each of the complex activities that we aim to classify can be composed into a set of simpler activities, with varying time durations. For example, patching the routers requires grasping a network cable, moving it towards the respective socket and pushing the cable until it is securely connected to the socket. Similarly, changing the printer cartridges requires pulling the cartridge out of the printer slot and then pushing the new cartridge into the printer slot. During the training phase, this activity structure is taken into account in a straightforward manner, simply by applying the same label to all windowed data collected for one activity. This is done automatically by our data gathering application while the participant performs an activity. In the classification phase, the performance of our system depends on the similarity between the complex activities. This can be expressed in terms of the similarity among the simple activities of which two complex activities are composed.

We can also observe that activity A8 is misclassified as A5. These activities are again similar in nature (hand movements involve inserting an object (cable or cartridge) inside a slot (Ethernet port or printer cartridge bay), as can be observed in Figure 6e,h. Adding beacon information drastically improves results for A4 and A8. Looking at Table 2, we can see that the locations of these activities are distinct compared to the rest of the activities, and beacon information helps the classifier discriminate the relevant data points. For example, A8 is no longer misclassified as A5. Although A5 and A8 can be both performed in Sector 2, the locations of the printers within this sector are, as one would expect, distinct from the locations where patching takes place.

Activities A5 and A7 also benefit from beacon data, but to a lesser degree. For example, A5 is still misclassified as A2 for more than 10% of the data. This is due to the fact that both activities take place in the same sector. Although this does not mean that their locations are exactly the same (in which case there would be no benefit from additional location information), they are not sufficiently different to result in greater classification improvement. We must also highlight the fact that increasing the window size improves by a small degree the performance of the LR classifier. As a small window size results in a more responsive activity recognition system (less waiting time to construct a data point), it is evident that LR suffers in that respect since for a window size of 1 s, the results are poor.

**Figure 12:**
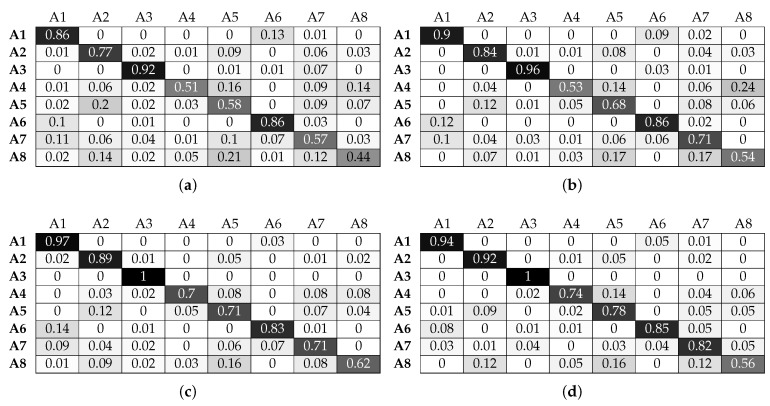
Normalised confusion matrices for KNN, with Wearable Feature Type 2, without beacon data. (**a**) *n* = 9, w = 1 s; (**b**) *n* = 10, w = 2 s ; (**c**) *n* = 8, w = 3 s; (**d**) *n* = 5, w = 4 s.

**Figure 13:**
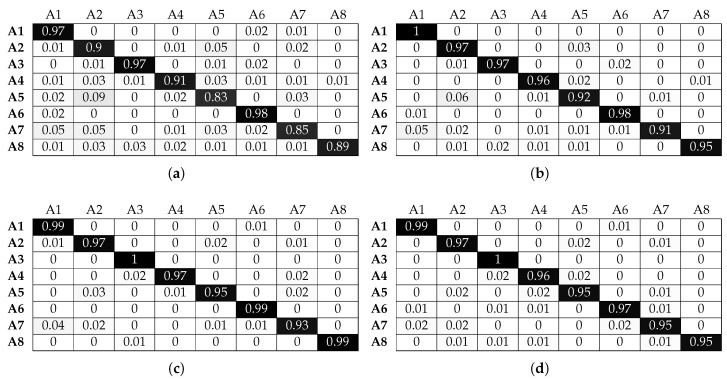
Normalised confusion matrices for KNN, with Wearable Feature Type 2, with beacon data. (**a**) *n* = 3, w = 1 s; (**b**) *n* = 3, w = 2 s ; (**c**) *n* = 3, w = 3 s; (**d**) *n* = 3, w = 4 s.

Figure 12 and Figure 13 illustrate the performance of the KNN classifier. We can observe that activities A1, A2, A3 and A6 are classified with over 90% accuracy without beacons, an improvement in performance compared to the LR classifier. Adding beacon data further improves performance, as expected. KNN benefits significantly from increasing the window size. This is shown for activities A4, A5 and A7 where for a 1-s window, they are below 60%. However, when the window size increases, they all reach an accuracy close to 75%, without beacon data. Adding beacon data further improves the performance of these activities. We must again note that, although these activities are performed in common sectors, the micro-locations inside each sector are different. For example, activity A7 (assembling the robot) and activity A5 (patching the router) take place on different parts of the workbenches inside the sectors. The KNN classifier can take advantage of this information to improve performance, something that the LR classifier could not achieve to the same degree. Finally, the benefit of location information is clearly shown in the case of activity A8. Without location information, the best accuracy obtained is 62%. With location information, it reaches 99% for the same window size (3 s).

**Figure 14:**
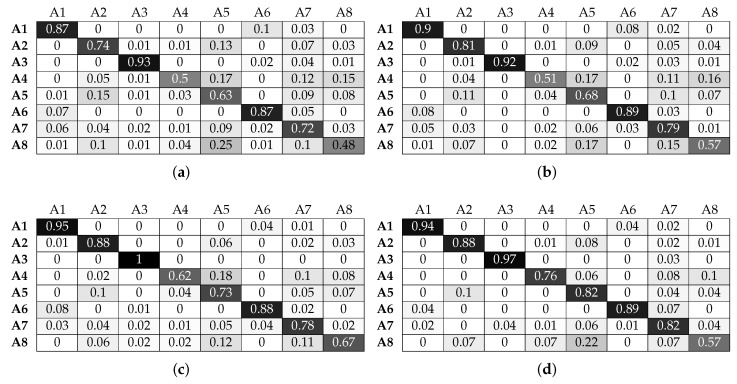
Normalised confusion matrices for random forest, with Wearable Feature Type 2, without beacon data. (**a**) 49, w = 1 s; (**b**) 50, w = 2 s ; (**c**) 43, w = 3 s; (**d**) 45, w = 4 s.

**Figure 15:**
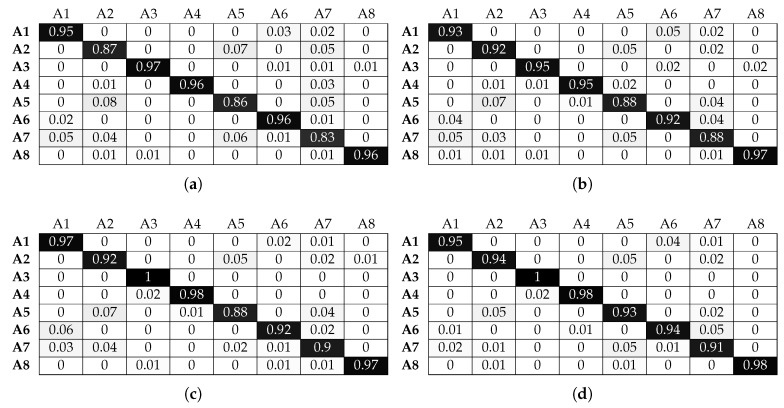
Normalised confusion matrices for random forest, with Wearable Feature Type 2, with beacon data. (**a**) *n* = 48, w = 1 s; (**b**) *n* = 46, w = 2 s ; (**c**) *n* = 42, w = 3 s; (**d**) *n* = 41, w = 4 s.

As seen in Figure 14 and Figure 15, the RF classifier exhibits a performance similar to KNN in terms of being able to take advantage of micro-location information. We can again confirm that adding beacon information improves drastically the classifier’s performance. Mores specifically, the classification accuracy for activities A4 and A8 (for their optimal window sizes) increases from 76 and 67% without beacon data to 98% for both activities when beacon features are fused with smart watch features. However, the RF classifier cannot fully take advantage of the feature fusion in the case of activities A5 and A7: the RF classifier cannot achieve accuracy above 93% and 91% when we use beacon information, and it only manages this for the maximum window size of 4 s.

**Figure 16:**
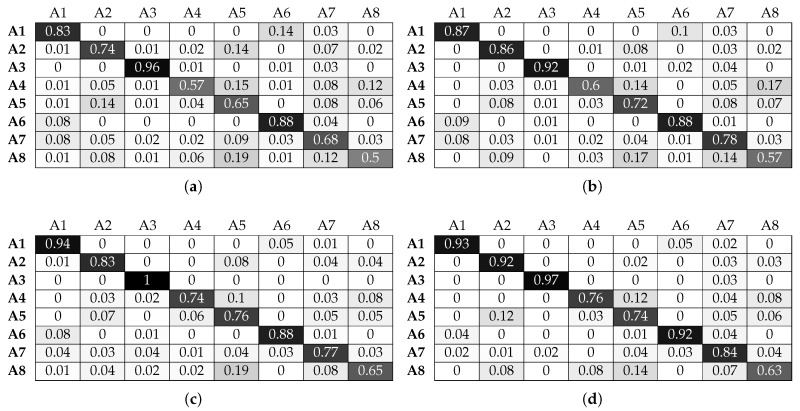
Normalised confusion matrices for SVM, with Wearable Feature Type 2, without beacon data. (**a**) C = 10, γ = 0.1, w = 1 s; (**b**) C = 10, γ = 0.1, w = 2 s; (**c**) C = 10, γ = 0.1, w = 3 s; (**d**) C = 10, γ = 0.1, w = 4 s.

**Figure 17:**
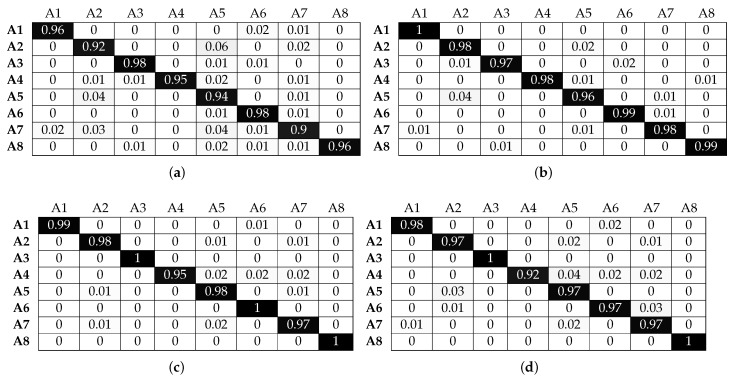
Normalised confusion matrices for SVM, with Wearable Feature Type 2, with beacon data. (**a**) C = 100, γ = 0.1, w = 1 s; (**b**) C = 10, γ = 0.1, w = 2 s; (**c**) C = 10, γ = 0.1, w = 3 s; (**d**) C = 10, γ = 0.1, w = 4 s.

Figure 16 and Figure 17 illustrate that SVM has the optimal recognition performance and also benefits the most from beacon information. More specifically, classification accuracy for activities A1, A2, A3 and A6 is above 85% without beacon data. This is further improved, as expected, when beacon information is used and reaches a classification accuracy of over 95%. Correctly classifying activities A4 and A5 proves more challenging since, as we explained above, both activities involve similar translational hand movement. However, SVM is the only classifier that reaches above 60% accuracy without beacon data for window sizes greater than 1 s. Adding beacon data increases the classification performance to near perfect accuracy levels. Furthermore, although SVM has a classification accuracy similar to that of the other classifiers for activity A7 without beacon information, it outperforms them with beacon information and reaches 97% accuracy. Looking more closely at the confusion matrices, we observe that activity A7 proves one of the most challenging activities to classify accurately for the other classifiers, even with beacon data. Although activity A7 takes place in the same set of sectors with activities A2, A5 and A5, the micro-locations inside each sector are different (i.e., location along a workbench). SVM can use this micro-location information, revealed by the beacon data, better than other classifiers, and this results in higher classification accuracy. The same behaviour is observed for activity A8: when using information solely from smart watches, classification accuracy does not reach a level above 65%. Adding beacon information results in perfect accuracy for most window sizes.

As a general note, we should highlight the fact that LR is a linear classifier, while KNN, RF and SVM are non-linear classifiers. When adding beacon data and increasing the dimensionality of our feature space, the data become non-linearly separable, and LR is not able to take advantage of the additional information. This results in worse classification performance compared to the other classifiers. We can also confirm this by inspecting Figure 7a and Figure 8a, where the gap in average $F_{1}score$ between LR and the other classifiers increases from 0.1 (without beacon data) to 0.15 (with beacon data).

## 7. Conclusions

In this work, we proposed an activity recognition framework for indoor environments, composed of off-the-shelf smart watches and BLE beacons. A mobile phone is responsible for gathering smart watch and beacon data and transmitting them to a server where the processing and classification takes place. Our approach uses location information revealed by the beacon data, to enhance the classification accuracy of the machine learning algorithms we employ. Our experimental results have shown that there is a clear improvement in the performance of our system when beacon data are used. However, the extent to which the location information can be advantageous depends on the type of classifier. LR cannot take full advantage of location information, while KNN and RF benefit more from the fusion of beacon data. SVM exhibits the highest performance gain when using beacon data. Furthermore, we observe that the more unique the location of an activity is with respect to the others, the higher the benefit in activity recognition performance. However, we must highlight that even subtle differences in activity locations are sufficient for a significant improvement in the classification accuracy (e.g., working on different parts of a workbench inside the same sector). Finally, location information can make the system more adaptive, as it allows for smaller window sizes, which results in less time required to collect and classify data.

In future work, we will further investigate human activities that can take place in an indoor setting, such as building emergency management [52,53]. This could prove beneficial for an emergency operation, as it could improve situational awareness with respect to the activities of building occupants in the instances before or after an incident took place. Finally, we will investigate a wider range of machine learning algorithms and consider the use of neural networks and deep learning for further improving our system’s performance.

## Figures and Tables

**Table 1:** HAR publication details.

Publication	Indoor Space			IMU Sensors			Classification Approach										COTS
	Home	Lab	Other	Acc	Mag	Gyro	SVM	KNN	LR	ANN	HMM	NB	DT	RF	CRF	Other	
[22]		X		X		X		X				X	X				X
[23]	X			X		X	X						X				
[25]		X		X			X	X		X		X	X	X			
[15]		X		X		X				X		X		X			X
[11]		X		X					X								
[16]	X			X		X							X				X
[28]		X		X												X	
[17]			X	X		X					X		X				X
[24]		X		X	X	X								X	X		
[18]		X		X			X		X			X	X				X
[20]	X			X							X				X		X
[26]		X		X	X	X	X			X		X					
[27]		X		X					X	X		X	X				
[14]		X		X			X	X				X	X	X			
[13]		X		X				X					X	X		X	X
[19]			X	X		X	X	X				X	X	X			X
[29]		X		X												X	

***Legend.*** IMU sensors: Acc, Accelerometer; Mag, Magnetometer; Gyro, Gyroscope; Classification approach: SVM, Support Vector Machines; KNN, k-Nearest Neighbours; LR, Logistic Regression; ANN, Artificial Neural Network; HMM, Hidden Markov Model; NB, Naive Bayes; DT, Decision Trees; RF - Random Forest; CRF, Conditional Random Field; COTS, Commercial-Off-The-Shelf.

**Table 2:** Activity codes.

Activity Code	Activity Name	Sector Codes
A1	Typing	S2, S3, S4
A2	Servicing	S2, S3, S4
A3	Scanning	S1
A4	Relocating	S1
A5	Patching	S2, S3, S4
A6	Installing	S1, S2, S3, S4
A7	Assembling	S2, S3, S4
A8	Refilling	S1, S2

## Abbreviations

The following abbreviations are used in this manuscript:

**ANN**	Artificial Neural Networks
**AP**	Access Point
**API**	Application Program Interface
**BLE**	Bluetooth Low Energy
**COTS**	Commercial-Off-The-Shelf
**CRF**	Conditional Random Field
**DT**	Decision Trees
**EP**	Emerging Pattern
**GNSS**	Global Navigation Satellite System
**HAR**	Human Activity Recognition
**HMM**	Hidden Markov Models
**IMU**	Inertial Measurement Unit
**IPS**	Indoor Positioning System
**NB**	Naive Bayes
**KNN**	K-Nearest Neighbours
**LR**	Logistic Regression
**MLP**	Multi-Layer Perceptron
**RF**	Random Forests
**RSSI**	Received Signal Strength Indicator
**SNR**	Signal-to-Noise Ratio
**SVM**	Support Vector Machines
**UUID**	Universally Unique Identifier
